# Intermittent Fasting Attenuates Obesity-Induced Triple-Negative Breast Cancer Progression by Disrupting Cell Cycle, Epithelial–Mesenchymal Transition, Immune Contexture, and Proinflammatory Signature

**DOI:** 10.3390/nu16132101

**Published:** 2024-07-01

**Authors:** Deok-Soo Son, Kaitlyn A. Done, Jubin Son, Michael G. Izban, Carlos Virgous, Eun-Sook Lee, Samuel E. Adunyah

**Affiliations:** 1 Department of Biochemistry, Cancer Biology, Neuroscience and Pharmacology, School of Medicine, Meharry Medical College, Nashville, TN 37208, USA; sadunyah@mmc.edu; 2 Biochemistry Program, College of Arts and Sciences, Spelman College, Atlanta, GA 30314, USA; 3 Neuroscience Program, College of Arts and Sciences, The University of Tennessee, Knoxville, TN 37996, USA; 4 Pathology, Anatomy and Cell Biology, Meharry Medical College, Nashville, TN 37208, USA; 5 Animal Core Facility, Meharry Medical College, Nashville, TN 37208, USA; 6 Department of Pharmaceutical Sciences, College of Pharmacy, Florida A&M University, Tallahassee, FL 32301, USA; eunsook.lee@famu.edu

**Keywords:** intermittent fasting, triple-negative breast cancer, obesity, epithelial–mesenchymal transition, proinflammatory signature

## Abstract

Obesity is associated with one-fifth of cancer deaths, and breast cancer is one of the obesity-related cancers. Triple-negative breast cancer (TNBC) lacks estrogen and progesterone receptors and human epidermal growth factor receptor 2, leading to the absence of these therapeutic targets, followed by poor overall survival. We investigated if obesity could hasten TNBC progression and intermittent fasting (IF) could attenuate the progression of obesity-related TNBC. Our meta-analysis of the TNBC outcomes literature showed that obesity led to poorer overall survival in TNBC patients. Fasting-mimicking media reduced cell proliferation disrupted the cell cycle, and decreased cell migration and invasion. IF decreased body weight in obese mice but no change in normal mice. Obese mice exhibited elevated plasma glucose and cholesterol levels, increased tumor volume and weight, and enhanced macrophage accumulation in tumors. The obesity-exacerbated TNBC progression was attenuated after IF, which decreased cyclin B1 and vimentin levels and reduced the proinflammatory signature in the obesity-associated tumor microenvironment. IF attenuated obesity-induced TNBC progression through reduced obesity and tumor burdens in cell and animal experiments, supporting the potential of a cost-effective adjuvant IF therapy for TNBC through lifestyle change. Further evidence is needed of these IF benefits in TNBC, including from human clinical trials.

## 1. Introduction

Obesity is a disease defined by excessive fat accumulation that impairs health and is rapidly increasing worldwide as a global epidemic, which is predicted to affect 1.12 billion adults by 2030, as stated by the World Health Organization [1]. Obesity is well known as a risk factor for many diseases, such as diabetes, cardiovascular disease, and cancer [2]. Several types of cancers are related to obesity, including pancreatic, endometrial, esophagus, rectal, kidney, liver, colon, gallbladder, breast, thyroid, and ovarian cancers [3]. In particular, one-fifth of cancer deaths are influenced by obesity, which in part induces chronic inflammation [4,5]. Obesity contributes to health disparity among ethnicities as the highest rates of obesity occur in African American (AA) women compared to other ethnic groups [6,7,8]. Breast cancer is the most general type of cancer and the second leading cause of cancer deaths in women. The five-year survival rate of breast cancer is 99% for the localized stage, 85.8% for the regional stage, and only 29% for the distant stage [9]. The subtypes of breast cancer are classified by the following immunohistochemical features: estrogen receptor (ER) positive (+), progesterone receptor (PR)+, and human epidermal growth factor receptor 2 (HER2) negative (−) luminal A (LA); ER+, PR+, and HER2+ luminal B (LB); ER-, PR-, and HER2+ HER2-enriched (HER2); ER-, PR-, and HER2- basal-like (BL) subtypes [10]. Triple-negative breast cancer (TNBC), which does not express ER, PR, and HER2, exhibits aggressive tumorigenicity and results in high mortality due to a lack of therapeutic targets compared to other subtypes [11]. Accordingly, numerous studies have been conducted to find alternative therapeutic approaches to treat TNBC. In addition to the high incidence rates of obesity, the health disparity is exacerbated by higher incidence rates of TNBC in AA women than in other ethnic groups [6,7,8], leading to poor survival rates [9]. Obesity is a risk factor for postmenopausal breast cancer, and abdominal obesity, known as central obesity, may increase the risk of TNBC [12]. Although breast cancer is one of the obesity-related cancers [3], the association between obesity and TNBC is still controversial. It is possible that survival rates could be improved in patients with obesity-related cancers if obese burdens are withdrawn.

Intermittent fasting (IF) utilizes time-restricted feeding that cycles between periods of fasting and eating, making it easier to follow than traditional calorie restriction. IF is known to have several health benefits, such as reduced obesity [5,13], improved immune function and insulin sensitivity [13,14], upregulated autophagy and mitophagy [15], lowered oxidative stress and inflammation [16,17,18], and increased longevity [19]. With regard to cancers, IF has been proposed to exert anti-cancer properties [20,21,22], improve the effectiveness of chemotherapy [21,23], reduce cancer incidence [22], support cancer prevention [16], and protect against chemotherapy toxicity [24]. Particularly, IF reduced the obesity burden and mammary tumor formation in animal models [25]; however, some studies showed no significant effects of IF on tumor size and body weight in mice bearing prostate cancer [26,27]. Therefore, further studies are necessary to identify IF-sensitive and resistant cancer types. Despite the adjuvant benefits of IF for treating cancer, the molecular mechanisms linking IF and cancer are not fully understood. Here, we investigated if obesity could affect TNBC progression and whether IF targets obesity-related TNBC using diet-induced obese and orthotopic mammary fat pad implantation models. The outcome of this study establishes fundamental evidence that IF can become a potentially cost-effective adjuvant therapeutic strategy for treating TNBC. Accordingly, IF may offer benefits to improve the efficacy of anticancer therapies and result in reduced side effects and the quality of life of patients with TNBC and other obesity-related cancers through lifestyle changes.

## 2. Materials and Methods

### 2.1. Meta-Analysis on Associations between Obesity and TNBC

The meta-analysis was performed based on the Preferred Reporting Items for Systematic Reviews and Meta-Analyses Statement [28]. Literature databases, such as PubMed, Cochrane Library, and Google Scholar, were explored from 2003 to 2023 with the following subject terms: obesity; TNBC; and overall survival. As published papers were reviewed, ethical approval and patient consent were not needed. The inclusion criteria were as follows: lean and obese female population in patients diagnosed with TNBC; risk ratio (RR) and the corresponding 95% confidence interval (95% CI) with overall survival (OS); and full-text English articles. The exclusion criteria were as follows: duplicated studies; reviews; case reports; abstracts; letters; obese male population; studies with insufficient data; animal studies; and non-English studies. In the case of reviews, the original research articles, which were mentioned in review papers, were checked for data analysis. Two independent investigators (DS and EL) extracted available data from eligible studies with input data verification (JS) for the validation of the findings, adjusting any discrepancies through discussion. The pooled RR and 95% CI were directly evaluated from each published study using Review Manager (RevMan 5.3; https://www.cochrane.org/; accessed on 1 December 2021) with the following criteria: (1) OS between the lean and obese female population in patients with TNBC and (2) obesity rate between ethnic groups in patients with TNBC. Heterogeneity between the included studies was measured by using Cochran’s Q test with *p*-value and *I*^2^ statistics. A *p*-value < 0.1 for Cochran’s Q test and *I*^2^ > 50% for the *I*^2^ test indicated significant heterogeneity among the included studies.

### 2.2. Cell Line and Cell Cultures

The human TNBC cell lines, MDA-MB-231 (ATCC^®^HTB-26™) and MDA-MB-468 (ATCC^®^HTB-132™), and mouse TNBC cell line PY8119 (ATCC^®^CRL-3278™) were purchased from the American Type Culture Collection (ATCC, Manassas, VA, USA). MDA-MB-231 and MDA-MB-468 cells were cultured at 37 °C in a water-saturated atmospheric air with Leibovitz’s L-15 Medium containing penicillin (100 I.U./mL)/streptomycin (100 μg/mL) and 10% FBS. PY8119 cells were cultured at 37 °C in a water-saturated atmosphere of 95% air and 5% CO_2_ with F-12K Medium containing penicillin/streptomycin and 5% FBS. After cellular attachment to the plates was confirmed under overnight culture, the medium was removed, and fresh Dulbecco’s Modified Eagle Medium (DMEM) was added as follows: complete media (CM, glucose: 4 g/L and 10% FBS) to generate intact TNBC cells and fasting-mimicking media (FM, low glucose: 1 g/L and 1% FBS) to generate fasted cells for 24–48 h, as described for conditions mimicking the metabolic consequences of fasting [29].

### 2.3. Cell Proliferation

Cell proliferation assays were carried out to measure color density using the cleavage of 3-(4,5-dimethylthiazol-2-yl)-2,5-diphenyltetrazolium bromide (MTT) to a colored product as described [30]. After a 48 h incubation for cell growth in a 24-well plate, each well was washed twice with phosphate-buffered saline (PBS) to remove the red phenol color of the medium, and then an MTT solution (1 mg/mL of phenol red-free media:PBS = 4:1) was added. The plates were incubated for 3 h under protection from light. The MTT solution was removed, and isopropanol was added into each well to dissolve the MTT color product. The plates were placed on a plate shaker and shaken gently for 10 min at room temperature. After no precipitates of MTT products were confirmed, optical density was measured at 595 nm using a microplate reader (Bio-Rad, Hercules, CA, USA). All experimental values were normalized to vehicle controls without cells.

### 2.4. Flow Cytometry

Cell cycle phases were examined by flow cytometry as described [31]. Briefly, cancer cells were seeded at equal densities and maintained in the culture for 24 h. Cells were then treated in triplicate with CM and FM for 48 h. The whole cells, including adherent and nonadherent cells, were harvested with cold PBS and fixed in cold 70% ethanol for at least 1 h at 4 °C. After being washed twice with cold PBS, fixed cells were resuspended with PBS, RNase A stock solution (10 µg/mL) was added, and incubated at 37 °C for 1 h, they were stained with propidium iodide (50 mg/mL in 0.1% (*w*/*v*) sodium citrate, 0.1% (*v*/*v*) Triton X-100) overnight at 4 °C. After overnight incubation, samples were washed twice with cold PBS, resuspended with PBS, and analyzed using a FACScan flow cytometer (BD Biosciences, Franklin Lakes, NJ, USA) and the percentage of the cells in G0/G1, S, and G2/M phases was quantified utilizing the FloJo 10.8 software (Tree Star Inc., Ashland, OR, USA).

### 2.5. Migration and Invasion

Cell migration or invasion assays were performed by seeding TNBC cells (2 × 10^5^ cells/mL in serum-free DMEM with 1% BSA) in the 24-well Transwell cell culture insert (Greiner Bio-one, VWR international, Radnor, PA, USA) or in a Matrigel (BD Biosciences, 1:3 diluted with PBS) coated Transwell system, respectively, as described [31]. The bottom chamber contained 0.5 mL CM and FM as a chemoattractant, respectively. After the plates were incubated for 24 h, the cells that remained inside the insert were removed with a cotton swab. Migrated or invaded cells on the filter were fixed with 3.7% formaldehyde, stained with 0.1% crystal violet, and washed with PBS to remove background staining. Digital images were captured with a BZ-X700 All-in-One Fluorescence Microscope (KEYENCE, Osaka, Japan). Quantitative analysis was performed using ImageJ 1.54f with purple selected as the color, followed by intensity comparison.

### 2.6. Diet-Induced Obese and Orthotopic Mammary Fat Pad Models

Mouse diet-induced obese and orthotopic mammary fat pad models were carried out under guidelines that were approved by the Institutional Animal Care and Use Committee at the Meharry Medical College (eProtocol #20-09-1034) and the National Institutes of Health (NIH) Guide for the Care and Use of Laboratory Animals. Four-week-old female mice (Strain #002216: B6.129S7-Rag1tm1Mom/J), which do not produce mature T cells or B cells, were purchased from Jackson Laboratory (Bar Harbor, ME, USA). The mice were kept in a specific pathogen-free animal facility at 22 °C ± 2 °C and 40%–60% humidity under a 12:12 light:dark cycle. The mice were fed with a normal diet (ND, 5% kcal from fat) and high-fat diet (HFD, D12492: 60% kcal from fat) purchased from Research Diets Inc. (New Brunswick, NJ, USA) for 9 weeks to produce normal and obese mice, respectively, as described [32]. We grouped mice as follows: (1) ND; (2) ND-IF; (3) HFD; (4) HFD-IF. After obese mice were confirmed using body weight gain (approximately 2-fold gain), MDA-MB-231 cells (5 × 10^5^ cells/mouse) in PBS/Matrigel (50:50) were injected into both 4th mammary fat pads. From the day following the orthotopic mammary fat pad injection of TNBC cells, alternate-day fasting (24 h fasting and feeding cycle) was applied in the IF groups. All mice in IF groups had free access to water during IF. The body weight of mice was measured weekly, and the health conditions of mice were monitored 3 times per week to assess the following parameters: hunched posture; lethargy and inactivity; impaired ambulation; shallow or labored breathing; hair coat condition; and change in the body weight. Mice showing signs of tumor burdens (~1200 mm^3^ of the tumor volume) and changes in appearance and activity were observed daily. When sluggish activity was observed, mice were euthanized for humane reasons. Tumor volume was calculated by the following formula: (length × width^2^)/2. The parameters of tumor potential were monitored as follows: (1) body and spleen weight; (2) tumor volume and weight; (3) proteomic chemokine/cytokine arrays in tumor tissues; (4) triglyceride, free fatty acid and cholesterol levels in sera; (5) immunoblots for cell cycle phase, autophagy, and epithelial–mesenchymal transition (EMT); (6) immune cell profiles in tumor tissues using H&E staining and immunohistochemistry.

### 2.7. Western Blots

Cell lysates and tumor tissues were prepared in a radio-immunoprecipitation assay buffer containing protease and phosphatase inhibitors (MilliporeSigma, St. Louis, MO, USA). After being incubated for 30 min on ice and vortexed several times, the obtained lysates and homogenized mixtures were centrifuged at 10,000× *g* for 10 min at 4 °C. Each clear supernatant, excluding the upper layer of lipid, was transferred to a new tube. Final protein lysates were prepared after additional centrifugation under the same conditions. The total protein concentration for lysates was determined by absorbance at 595 nm using the Bio-Rad protein assay (Bio-Rad, Hercules, CA, USA). The protein lysates were fractionated on SDS-polyacrylamide gels and transferred to polyvinylidene difluoride membranes based on previously established procedures [33]. Antibodies for Akt (#4060/4691), MAPK (#9910/9926), Autophagy (#4445), EMT (#9782), and Cell Cycle Phase (#17498) were obtained from Cell Signaling Technology (Danvers, MA, USA). The β-actin (C4) antibody (Santa Cruz Biotechnology, Santa Cruz, CA, USA) served as an internal loading control. The targeted protein bands were visualized by chemiluminescence detection kits (MilliporeSigma, Burlington, MA, USA).

### 2.8. Biochemical Analyses in Plasma

Plasma levels of triglyceride, free fatty acid, and cholesterol (total, HDL, and LDL/VLDL) were measured by EnzyChrom™ Triglyceride (ETGA-200), Free Fatty Acid (EFFA-100), Total, HDL, and LDL/VLDL (E2HL-100) Cholesterol Assay Kits (BioAssay Systems, Hayward, CA, USA), according to the manufacturer’s instructions. The optical density of each well was evaluated using a microplate reader at a 570 nm wavelength. Glucose levels were measured by the Glucose Colorimetric Assay Kit (Cayman Chemical, Ann Arbor, MI, USA). The absorbance of each well was determined using a microplate reader at 514 nm wavelength.

### 2.9. Histological and Immunohistochemical Evaluation of Tumor Tissues

Tumor samples were analyzed using routine H&E stain and immunohistochemistry as described [32]. Paraffin slide sections were immersed three times in xylenes, followed by rehydration in a series of twice alcohol washes (100% and 95%, respectively). For heat antigen retrieval, sections were treated for 15 min in boiling EDTA solution (1 mM, pH 8.0). After being blocked in goat serum for 1 h, sections were incubated overnight with specific primary antibodies as follows: vimentin (D21H3) for EMT; F4/80 (D2S9R) for Mφ; Ly-6G (E6Z1T) for neutrophils; arginase-1 (D4E3M) for MDSCs, CD11c (D1V9Y) for dendritic cells; and CD8α (D4W2Z) for CD8 T cells (Cell Signaling Tech., Danvers, MA, USA). Sections were incubated for 1 h with the secondary antibody matched to the primary antibody and developed using SignalStain^®^ DAB Substrate kit (#8059; Cell Signaling Technology, Danvers, MA, USA), followed by hematoxylin counterstain. Digital images of slides were captured using a BZ-X700 All-in-One Fluorescence Microscope (KEYENCE).

### 2.10. Proteomic Array for Cytokine and Chemokine Signature

Cytokine and chemokine signatures in tumor tissues were evaluated using Proteome Profiler Mouse Cytokine Array (ARY006; R&D Systems, Minneapolis, MN, USA), according to the manufacturer’s instructions, as described previously [32]. Spot intensities were calculated with ImageJ (https://imagej.net; accessed on 25 July 2023) by subtracting the average background signals and normalizing them with reference spots.

### 2.11. Statistical Analysis

The Fiji software (https://imagej.net; accessed on 25 July 2023) was used to process and analyze scientific images [34]. Data were analyzed using the paired Student’s *t*-test and one-way analysis of variance (ANOVA), as appropriate. When a statistical significance (*p* < 0.05) was determined by ANOVA, the data were further analyzed by Tukey’s pairwise comparisons to detect specific differences between treatments. R-squared values for correlations between continuous variables were calculated from linear regression, utilizing the Data Analysis Tools in MS Excel 2021 (Microsoft 365). Differences in total tumor volumes were evaluated by the log–rank test.

## 3. Results

### 3.1. Obesity Is Associated with Poor Overall Survival of Women with TNBC

**Figure 1:**
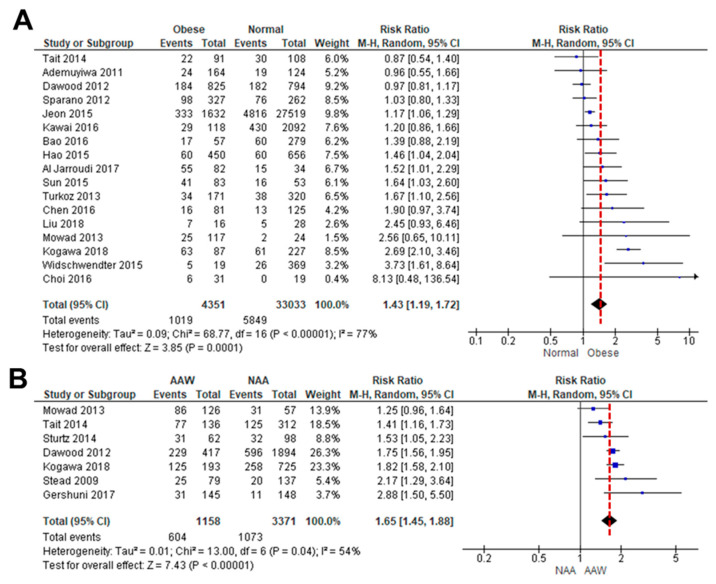
Meta-analysis for the association between obesity and TNBC. (**A**) Forest plot for a risk ratio on OS between obese and lean patients with TNBC. Seventeen cohort studies with a total sample size of 37,384 participants met the eligibility criteria to be included in quantitative analysis, with an ascending order. (**B**) Forest plot for a risk ratio of obesity between AA (AAW) and non-AA women (NAA) in the incidence of TNBC. Seven cohort studies with a total sample size of 4529 participants were included in quantitative analysis, with an ascending order. Patients with normal and obese weights were considered as <25 of BMI and >30 of BMI, respectively [8,35,36,37,38,39,40,41,42,43,44,45,46,47,49,51,52,53,54,55].

As the association between obesity and TNBC is controversial, we performed a systematic review of how obesity correlates with OS in TNBC patients [35,36,37,38,39,40,41,42,43,44,45,46,47,48,49,50,51,52,53]. A meta-analysis of OS data between lean and obese TNBC patients was available in 17 studies. Because a high level of heterogeneity (*I*^2^ = 77%, *p* < 0.00001) was observed between the studies, a random-effects model was used for the analysis (Figure 1A). Obesity led to poorer OS in TNBC patients (HR: 1.43, 95% CI: 1.19–1.72) than in lean patients (Figure 1A). We also analyzed how obesity might influence the incidence of TNBC between AA and non-AA women [8,35,36,40,43,54,55]. A meta-analysis of TNBC incidence data between obese AA and non-AA women was available in six studies. A random-effects model was used based on heterogeneity (*I*^2^ = 54%, *p* = 0.04). Obesity was associated with a higher risk of TNBC incidence in AA women (HR: 1.65, 95% CI: 1.45–1.88) compared to non-AA women (Figure 1B). These results indicated that obesity leads to a higher risk of TNBC in AA women compared to non-AA women, probably accelerating obesity-derived TNBC progression.

### 3.2. Fasting-Mimicking Condition Attenuates Progression-Associated Properties of TNBC Cells

We determined the effects of cell culture growth conditions that mimic the metabolic consequences of fasting on progression-associated properties of TNBC-derived cell lines, such as cell proliferation, migration, and invasion. Human MB-231 and MB-468 cells are known to have mesenchymal-like and basal TNBC characteristics, respectively [56]. PY8119 cells are a mouse TNBC cell line with mesenchymal-like characteristics.

**Figure 2:**
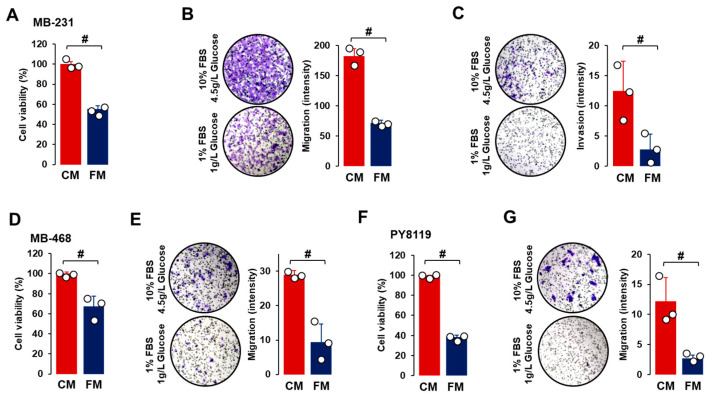
Comparison between CM and FM in TNBC progression. (**A**–**C**) Comparative effects of CM and FM on cell proliferation, cell migration, and cell invasion in MB-231 cells, respectively. (**D**,**E**) Comparative effects of CM and FM on cell proliferation and migration in MB-468 cells, respectively. (**F**,**G**) Comparative effects of CM and FM on cell proliferation and migration in PY8119 cells, respectively. Cells were incubated for 48 h for cell proliferation and 24 h for cell migration and invasion in each cell line. A cell proliferation assay was performed using MTT, and values were normalized to controls without cells. Cell migration and invasion assays were conducted in Matrigel-free and -coated Transwell systems, respectively. Quantitative analysis of intensity was performed using ImageJ through the color threshold, followed by particle analysis. Experiments were performed in triplicate (n = 3), and all data are shown as mean ± standard deviation (SD). # *p* < 0.05 in each group calculated by the paired Student’s *t*-test. CM: complete conditioned media with 4 g/L glucose and 10% FBS; FM: fasting-mimicking conditioned media with 1 g/L glucose and 1% FBS.

For the cell viability assay, TNBC cells were grown for 48 h in CM or FM to allow for enough cell growth doubling time. Compared to CM, FM decreased cell proliferation in all TNBC cell lines (Figure 2A,D,F), indicating that the fasting-mimicking condition contributed to reduced tumor growth. Cell migration was also reduced after FM treatment compared to CM in all TNBC cell lines (Figure 2B,E,G). Under CM growth conditions, cell invasion properties were observed in MB-231 cells but not in MB-468 and PY8119 cells. FM attenuated cell invasion in MB-231 cells compared to CM treatment (Figure 2C). FM-induced reduction in cell migration and invasion indicated that a fasting-mimicking condition plays a role in blocking cancer metastasis, probably by disrupting EMT-related signaling.

### 3.3. The Fasting-Mimicking Condition Disrupts TNBC Cell Cycle

**Figure 3:**
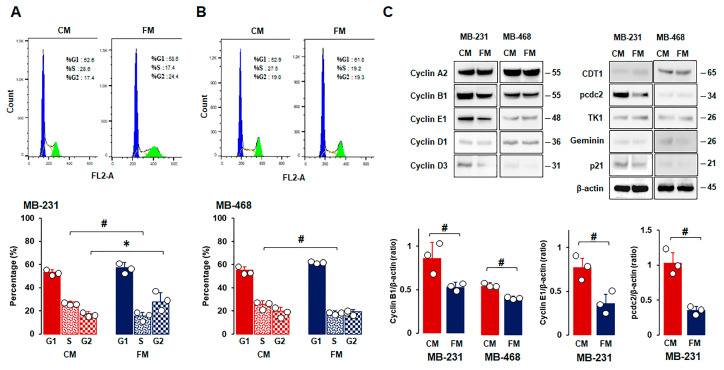
Comparison between CM and FM in cell cycle stages and phase-related proteins in TNBC cells. (**A**,**B**) Flow cytometry analysis between CM and FM on cell cycle in MB-231 and MB-468 cells, respectively. Cells were treated with CM and FM for 24 h. Flow cytometry assays were performed to determine the percentage of cells in each phase. Representative histograms are shown. (**C**) Comparative effects of CM and FM on the cell cycle phase-related protein expressions in MB-231 and MB-468 cells, respectively. Whole-cell lysates were prepared, and Western blots were carried out using antibodies specific to cell cycle phase-related proteins. β-actin was used as a loading control. Representative pictures are shown. Experiments were performed at least in triplicate (n = 3), and all data are shown as mean ± SD. * and # *p* < 0.05 in each group calculated by the paired Student’s *t*-test. CM: complete conditioned media with 4 g/L glucose and 10% FBS; FM: fasting-mimicking conditioned media with 1 g/L glucose and 1% FBS; CDT1: cell division control protein 10 (cdc10)-dependent transcript 1 protein; pcdc2: phospho-cdc2; TK1: thymidine kinase 1.

As FM attenuated cell proliferation (Figure 2A,D,F), we used flow cytometry to examine the cell cycle profiles of TNBC cells under CM and FM. Flow cytometry analysis revealed that FM decreased the S phase in all TNBC cells compared to CM (Figure 3A,B). FM increased the G2 phase in MB-231 cells (Figure 3A,C), but there was no significant increase in MB-468 cells with the increased trend in the G1 phase compared to CM (Figure 3B). These results indicated that the fasting-mimicking condition differentially disrupts the cell cycles in a TNBC subtype-specific manner, requiring further study to clarify fasting-induced cell cycle profiles between TNBC subtypes. Cell phase-regulatory protein levels in MB-231 and MB-468 cells were analyzed to further evaluate fasting-derived effects on cell proliferation. MB-231 cells had high levels of cyclin A2, cyclin B1, cyclin E1, and pcdc2, while MB-468 cells had high levels of CDT1, cyclin A2, and cyclin B1 (Figure 3C). FM reduced expression levels of cyclin B1, cyclin E1, and pcdc2 in MB-231 cells and cyclin B1 in MB-468 cells compared to CM (Figure 3C). These results indicated that a fasting-mimicking condition deregulates cell phase-related proteins, in particular by decreasing cyclin B1 protein levels, which is required to control the G2/M transition properly, followed by reduced cell division.

### 3.4. Fasting-Mimicking Condition Targets EMT in TNBC Cells

**Figure 4:**
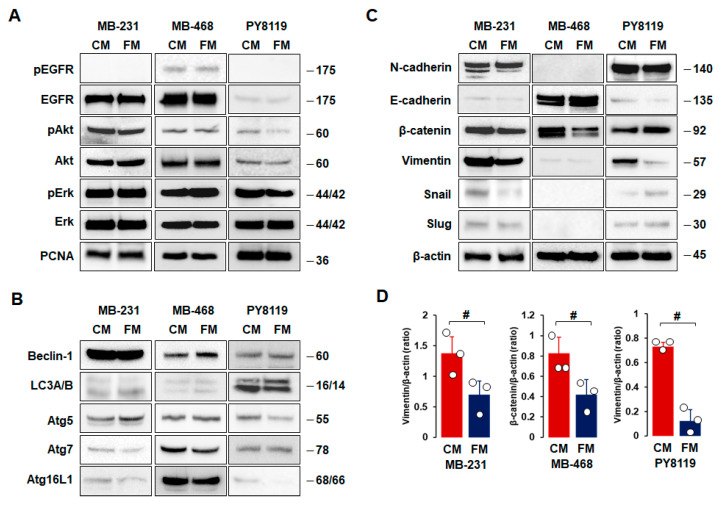
Comparison between CM and FM on cell survival, autophagy, and EMT in TNBC cells. (**A**–**C**) Comparative effects of CM and FM on protein expression levels related to cell survival, autophagy, and EMT in MB-231, MB-468, and PY8119 cells, respectively. Cells were treated with CM and FM for 24 h. Whole-cell lysates were prepared, and Western blots were carried out using antibodies specific to cell survival-, autophagy-, and EMT-related proteins. β-actin was used as a loading control. Representative pictures are shown. (**D**) A significant difference between CM and FM on vimentin and β-catenin expression levels in MB-231, MB-468, and PY8119 cells. Experiments were performed at least in triplicate (n = 3), and all data are shown as mean ± SD. # *p* < 0.05 in each group as calculated by the paired Student’s *t*-test. CM: complete conditioned media with 4 g/L glucose and 10% FBS; FM: fasting-mimicking conditioned media with 1 g/L glucose and 1% FBS; EGFR: epidermal growth factor receptor; pEGFR: phospho-EGFR; PCNA: proliferating cell nuclear antigen; LC3A/B: light chain 3A/B.

As FM attenuated cell proliferation, migration, and invasion (Figure 2), we compared the effects of CM and FM on cell survival, autophagy, and EMT in TNBC cells. There were no significant differences between CM and FM on cell survival- and proliferation-related proteins, such as EGFR, Akt, Erk, and PCNA, in all TNBC cells (Figure 4A). In addition, we found no significant differences between CM and FM on autophagy-related proteins, such as beclin-1, LC3A/B, Atg5, Atg7, and Atg16L1, in all TNBC cells (Figure 4B). MB-231 and PY8119 cells showed high levels of N-cadherin, β-catenin, and vimentin, while MB-468 cells had high levels of E-cadherin and β-catenin (Figure 4C). These results confirmed mesenchymal-like characteristics in both MB-231 and PY8119 cells but basal-like characteristics in MB-468 cells. Statistical analysis revealed that FM decreased expression levels of vimentin in both MB-231 and PY8119 cells and decreased β-catenin protein levels in MB-468 cells compared to CM (Figure 4D). FM tended to increase E-cadherin levels in MB-468 cells but with no significance (Figure 4C). On the other hand, slug and snail protein levels were lower in TNBC cells compared to other EMT proteins (Figure 4C). FM-induced reduction in vimentin indicated that the fasting-mimicking condition plays a pivotal role in metastasizing TNBC cells through EMT alteration. Overall, EMT-related proteins appear more responsive to the fasting-mimicking condition than cell survival- and autophagy-related proteins.

### 3.5. IF Attenuates Tumor Burdens in TNBC Progression

**Figure 5:**
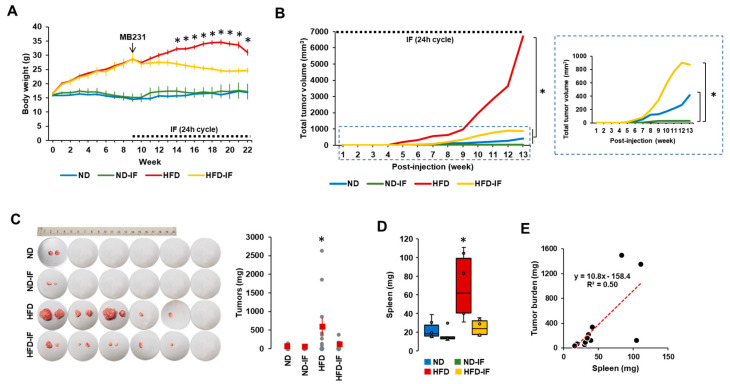
The tumor potential profiles of TNBC in ND, ND-IF, HFD, and HFD-IF mice. (**A**) Body weight trends between ND, ND-IF, HFD, and HFD-IF mice treated with human MB-231 TNBC cells. After confirming a body weight gain at 9 weeks (n = 6/group), MB-231 cells were injected into both 4th mammary fat pads. Alternate-day fasting (24 h fasting and feeding cycle) was applied in fasting groups. * *p* < 0.05 between non-IF and IF in each ND and HFD group as calculated by the paired Student’s *t*-test. (**B**) Total tumor volume among ND, ND-IF, HFD, and HFD-IF mice. Dot square indicates magnificent graph for total tumor volume among ND, ND-IF, HFD, and HFD-IF mice. * *p* < 0.05 as calculated by the log–rank test. (**C**) Tumor burden among ND, ND-IF, HFD, and HFD-IF mice. (**D**) Spleen weight between ND, ND-IF, HFD, and HFD-IF mice by boxplots. * *p* < 0.05 between groups as analyzed by ANOVA and Tukey’s pairwise comparison tests. (**E**) Correlation of tumor and spleen weights. R-squared values were calculated from linear regression using the Data Analysis Tools in MS Excel. IF: intermittent fasting of 24 h cycle.

Based on in vitro data showing FM-induced reduction in TNBC progression (Figure 2, Figure 3 and Figure 4), we performed in vivo experiments to determine the therapeutic benefits of fasting on TNBC using diet-induced obese and orthotopic mammary fat pad implantation models. After confirming body weight gain in HFD-fed mice, we employed 24-h cycle IF to induce fasting conditions. IF decreased body weight in HFD-fed mice but not in ND-fed mice (Figure 5A). For several weeks after 24 h cycle IF, ND-IF mice tended to gain body weight due to increased food consumption after starvation compared to ND mice. These results indicate that IF has more benefits in the obese population compared to the normal-weight population. As tumor volumes varied between each group, we calculated total tumor volumes followed by the log-rank test for statistical significance. Total tumor volumes were observed in the following order: HFD > HFD-IF = ND > ND-IF mice (Figure 5B). Tumor mass was palpable earlier in HFD mice than in other groups (Figure 5B), indicating that obesity accelerates TNBC progression. IF slowed down TNBC progression in both HFD- and ND-fed mice (Figure 5B), suggesting that IF may provide therapeutic adjuvant benefits for TNBC in both obese and normal conditions. Ratios of tumor development for each group were as follows: one out of six mice for the ND and ND-IF groups and five out of six mice for the HFD and HFD-IF groups (Figure 5C). These results suggest that obesity may promote tumorigenesis. Tumor weight was higher in HFD mice compared to other groups (Figure 5C) and correlated with total tumor volume (Figure 5B). Spleen weight exhibited a similar pattern as described in tumor weight, being higher in HFD mice compared to other groups (Figure 5D). Spleen weight positively correlated with tumor weight (R^2^ = 0.50) (Figure 5E). As with tumor burden, IF also attenuated obesity-induced splenomegaly (Figure 5D). Spleen enlargement associated with tumor burden is also observed in patients with breast cancer [57].

### 3.6. IF Lowered Obesity-Elevated Levels of Glucose and Cholesterol in Plasma

**Figure 6:**
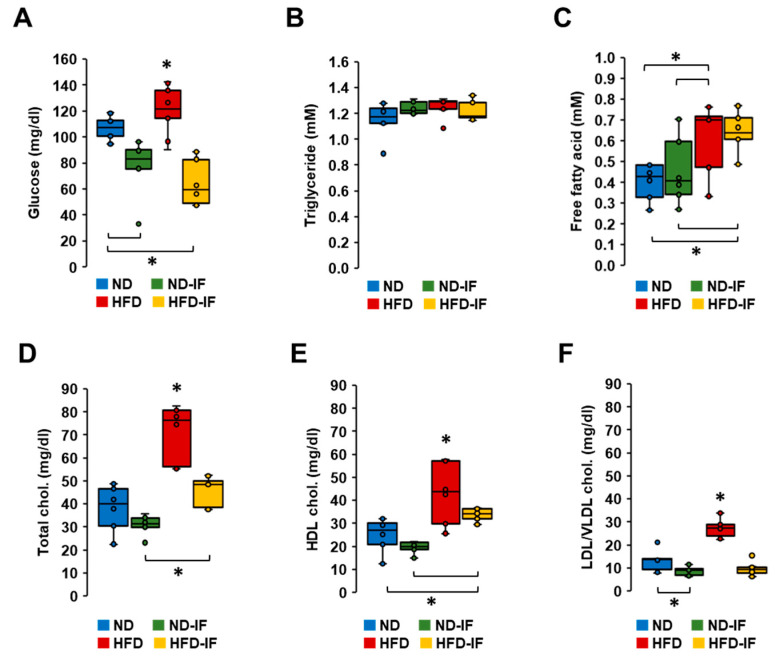
Biochemical characteristics of TNBC-derived sera in diet-induced obese and orthotopic mammary fat pad models. (**A**) Glucose, (**B**) triglyceride, (**C**) free fatty acid, (**D**) total cholesterol, (**E**) HDL cholesterol, (**F**) LDL/VLDL cholesterol levels in TNBC-derived sera of ND, ND-IF, HFD, and HFD-IF mice (n = 6/each group) using quantitative colorimetric assays with duplicate measurements. Box plots were graphed from MS Excel. * *p* < 0.05 between groups as analyzed by ANOVA and Tukey’s pairwise comparison tests. IF: intermittent fasting of 24 h cycle; HDL: high-density lipoprotein; LDL: low-density lipoprotein; VLDL: very-low-density lipoprotein.

As IF attenuated TNBC progression (Figure 5), we examined which systemic parameters were responsive to IF by measuring plasma levels of glucose, triglycerides, free fatty acids, and cholesterols. HFD-fed mice showed higher levels of glucose compared to those from other groups (Figure 6A). As expected, IF reduced glucose levels in both normal and obese conditions (Figure 6A). Interestingly, plasma levels of triglycerides between groups were not statistically different (Figure 6B). Although plasma levels of free fatty acids were higher in HFD-fed mice compared to ND-fed mice, IF had no effect on their overall levels in either ND-fed or HFD-fed mice (Figure 6C). On the other hand, plasma levels of total, HDL, and LDL/VLDL cholesterol levels were higher in HFD-fed mice compared to those from other groups (Figure 6D–F). IF inhibited obesity-induced levels of total, HDL, and LDL/VLDL cholesterols (Figure 6D–F). However, IF lowered LDL/VLDL cholesterol levels in the normal condition without altering total and HDL cholesterol levels (Figure 6D–F). Overall, IF appears to play a role in regulating glucose and cholesterol levels.

### 3.7. IF Attenuated Obesity-Induced Lipid Droplets and Vimentin Levels in Tumor Tissues

**Figure 7:**
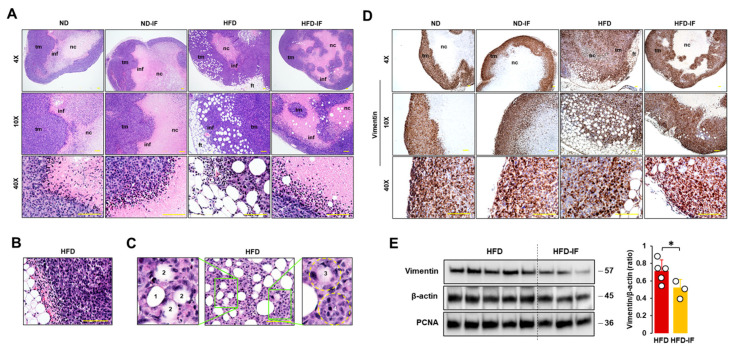
Histological evaluation of tumor tissues in diet-induced obese and orthotopic mammary fat pad models. (**A**) Histological features of tumor tissues in ND, ND-IF, HFD, and HFD-IF mice using H&E stain. (**B**) Abundant blood vessels between fat and tumor tissues in HFD-fed mice. (**C**) Invasive tumor cells into lipid droplets in HFD-fed mice. 1: intact lipid droplet, 2: initial invasion of tumor cells, 3: deeper invasion of tumor cells, 4: ball shape to show occupancy of tumor cells in lipid droplet. (**D**) Disposition of vimentin-positive cells in tumor tissues from ND, ND-IF, HFD, and HFD-IF mice using immunohistochemistry. (**E**) Comparative effects of HFD and HFD-IF on vimentin expression levels in tumor tissues. Tumor lysates were prepared, and Western blots were carried out using antibodies specific to vimentin and PCNA. β-actin was used as a loading control. All data are shown as mean ± SD. * *p* < 0.05 in each group as calculated by the paired Student’s *t*-test. IF: intermittent fasting of 24 h cycle; tm: tumor tissue; ft: fat tissue; inf: tumor-infiltrating immune cells; nc: necrotic region.

We evaluated histological features of tumor tissues in diet-induced obese and orthotopic mammary fat pad models. Tumor masses in HFD-fed mice had thicker tumoral cell layers and more abundant adipocytes as compared to other groups (Figure 7A). Tumor masses showed infiltrating immune cells between tumoral and necrotic regions (small hematoxylin dense cells in Figure 7A). Regions between adipose and tumor tissues also showed evidence of recruited immune cells and abundant blood vessels in HFD-fed mice (Figure 7A,B). Tumor cells seemed to invade into obesity-induced lipid droplets in tumor tissues by the following process: intact lipid droplet (Figure 7C1); initial invasion of tumor cells (Figure 7C2); deeper invasion of tumor cells (Figure 7C3); and finally invasion of tumor cells into lipid droplet (Figure 7C4). Since the fasting-mimicking condition inhibited expression levels of vimentin protein as an EMT marker in MB-231 cells (Figure 4C), we evaluated vimentin-positive cells in tumor tissues. Vimentin-positive cells showed intensively at the edges of tumor tissues and rapidly developed tumor growth in zones between adipose and tumor tissues (Figure 7D). A comparison of vimentin protein levels in lysates from tumor tissues between HFD and HFD-IF groups revealed that IF diminished obesity-induced vimentin expression, but PCNA protein levels were no different in these two groups (Figure 7E). These results are consistent with our cell model data that the fasting-mimicking condition reduced vimentin protein levels but showed no significant change in PCNA protein levels (Figure 4A,C).

### 3.8. IF Diminished Obesity-Induced Accumulation of Mφ in the Immune Cell Contexture of Tumor Tissues

**Figure 8:**
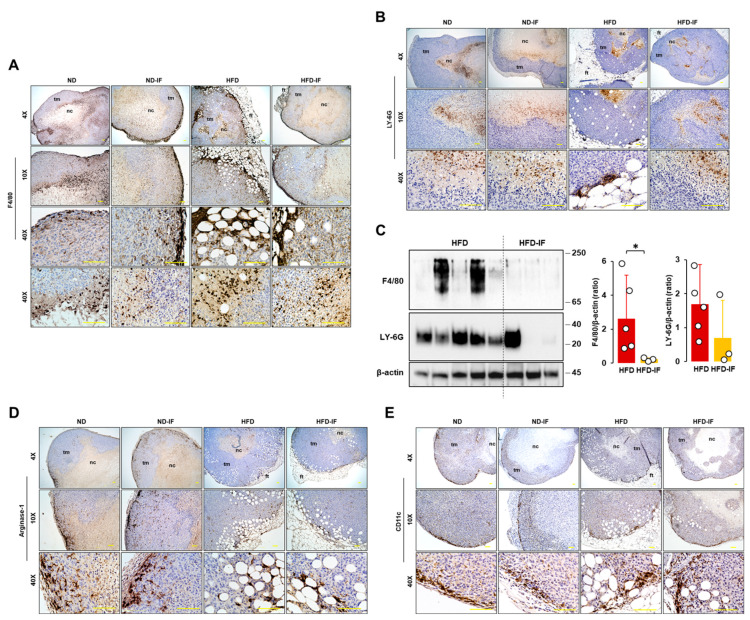
Immunohistochemical evaluation of immune cells in tumor tissues from diet-induced obese and orthotopic mammary fat pad models. Histological features of (**A**) F4/80 and (**B**) LY-6G-positive cells in tumor tissues from ND, ND-IF, HFD, and HFD-IF mice. (**C**) Comparative effects of HFD and HFD-IF on F4/80 and LY-6G expression levels in tumor tissues. Tumor lysates were prepared, and Western blots were carried out using antibodies specific to F4/80 and LY-6G. β-actin was used as a loading control. All data are shown as mean ± SD. * *p* < 0.05 in each group as calculated by the paired Student’s *t*-test. Histological features of (**D**) arginase- and (**E**) CD11c-positive cells in tumor tissues from ND, ND-IF, HFD, and HFD-IF mice. IF: intermittent fasting of 24 h cycle; tm: tumor tissue; ft: fat tissue; nc: necrotic region.

Because tumor-infiltrating immune cells affect clinical outcomes, we evaluated immune cell infiltration in tumor tissues. F4/80 staining as an Mφ marker appeared intensively at the edges of tumor tissues and in regions between adipose and tumor tissues (Figure 8A). Furthermore, F4/80-positive cells were shown intensively in close areas between tumoral and necrotic regions (Figure 8A). On the other hand, LY-6G staining as a neutrophile marker appeared intensively in the necrotic area and in some regions between adipose and tumor tissues (Figure 8B). LY-6G-positive cells appeared broadly inside the necrotic area, far away from the nearby area between tumoral and necrotic regions (Figure 8B), contrasting with F4/80-positive cells located in tumor tissues (Figure 8A). Comparison of F4/80 and LY-6G levels in lysates from tumor tissues between HFD and HFD-IF groups revealed that IF decreased obesity-induced F4/80 expression levels but had no effect on LY-6G levels (Figure 8C). Myeloid-derived suppressor cells (MDSCs) express arginase-1 highly and are recruited to tumor tissues [58]. Most arginase-1-positive cells appeared sporadically around the edges of tumor tissues to trigger tumor growth and some regions between adipose and tumor tissues (Figure 8D). CD11c-positive cells, as a dendritic cell marker, appeared intensively at the edges of tumor tissues to trigger tumor growth rather than regions between adipose and tumor tissues (Figure 8E). There were no changes in arginase-1 and CD11c protein levels in tumor lysates between HFD and HFD-IF. CD4- and CD8-positive cells were not found in tumor tissues because of T cell-deficient mice.

### 3.9. IF Attenuated Obesity-Induced Cyclin B1 Levels and Inflammatory Conditions in Tumor Tissues

**Figure 9:**
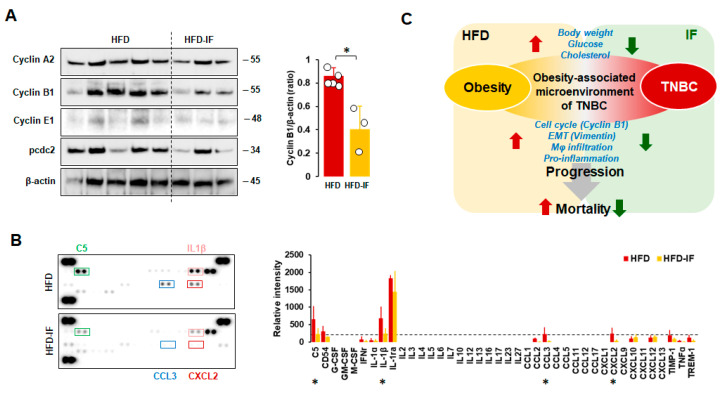
Comparison between HFD and HFD-IF of the cell cycle and cytokine signature. (**A**) Comparative effects of HFD and HFD-IF on cell cycle-related protein expression levels in tumor tissues. Tumor lysates were prepared, and Western blots were carried out using antibodies specific to cell cycle phase-related proteins. β-actin was used as a loading control. Column graph indicates a significant difference between HFD (n = 5) and HFD-IF (n = 3) on cyclin B1 expression levels. All data are shown as mean ± SD. * *p* < 0.05 in each group as calculated by the paired Student’s *t*-test. (**B**) Cytokine signatures of tumor tissues in HFD and HFD-IF mice by proteomic array. Relative intensity of spots to express cytokine levels was calculated by ImageJ. All data are shown as mean ± SD. Total spots from HFD and HFD-IF are n = 10/protein and n = 6/protein, respectively. * *p* < 0.05 in each group as calculated by the paired Student’s *t*-test in samples above the threshold value (relative intensity = 200, dot line). Representative pictures were selected from among tumor tissues from HFD and HFD-IF mice. C5: complement component 5; CD54: cluster of differentiation 54 or intercellular adhesion molecule 1 (ICAM-1); G-CSF: granulocyte colony-stimulating factor; GM-CSF: granulocyte-macrophage CSF; M-CSF: macrophage CSF; IFNγ: interferon-gamma; IL-1ra: interleukin-1 receptor antagonist; CCL: C-C motif chemokine ligand; CXCL: C-X-C motif chemokine ligand; TIMP1: tissue inhibitor matrix metalloproteinase 1; TNFα: tumor necrosis factor α; TREM-1: triggering receptor expressed on myeloid cells. (**C**) Schematic for inhibitory effects of IF on HFD-induced TNBC progression.

Because the fasting-mimicking condition inhibited cell cycle-related proteins in MB-231 cells (Figure 3C), we evaluated expression levels of cell cycle-related proteins in tumor tissues from HFD and HFD-IF mice. IF decreased obesity-induced cyclin B1 protein levels but showed no changes in cyclin A2, cyclin E2, and pcdc2 protein levels (Figure 9A). These results support our cell model data that the fasting-mimicking condition reduced cyclin B1 protein levels (Figure 3C). Consequently, we evaluated if IF could influence cytokine signatures in tumor tissues from HFD and HFD-IF mice using proteomic arrays. Tumor tissues from HFD-IF mice diminished C5, IL-1β, CCL3, and CXCL2 protein levels compared to those from HFD mice (Figure 9B), indicating that IF attenuates obesity-induced inflammatory conditions in the tumor microenvironment.

## 4. Discussion

A significant finding of this study is that IF attenuates obesity-induced TNBC progression involving multiple pathways, such as disrupted cell cycles, diminished EMT, systemic reduction in glucose and cholesterol levels, decreased Mφ infiltration into the tumor immune contexture, and downregulated inflammatory factors in the tumor microenvironment. Although there is controversy concerning the association between obesity and TNBC [35,36,37,38,39,40,41,42,43,44,45,46,47,48,49,50,51], a systematic review of 17 cohort studies revealed that obesity leads to poor OS in patients with TNBC (Figure 1A), being supported by other reviews [48]. Furthermore, obese AA women showed a higher risk of TNBC compared to non-AA women (Figure 1A), indicating health disparities in the vulnerability of obesity and TNBC. These results are consistent with other reviews that AA women are more likely to be obese and to be diagnosed with TNBC compared to non-Hispanic white women [6,59]. Even obese Caucasian women are prevalent in TNBC compared to non-TNBC [60]. Central obesity with a high waist/hip ratio showed poor OS in TNBC, serving as an independent prognostic factor for TNBC [41]. Thus, lifestyle interventions, such as IF, dietary alteration, and physical activity, appear as promising candidates to target the obesity–TNBC axis through reduced obesity burdens. On the other hand, a systematic review revealed that physical activity was associated with reduced breast cancer deaths in patients with ER-positive tumors but no significant effects in patients with ER-negative tumors [61], indicating the minor beneficial effects of physical activity in TNBC mortality.

Our TNBC results showed that IF could be more effective in the obese condition compared to the normal condition, decreasing body weight and tumor volume/weight (Figure 5A). Consistently, IF of the 5:2 day cycle decreased body weight in HFD-fed rats but not in ND-fed rats [62]. The fasting-mimicking diet (FMD) was also found to reduce body weight and tumor volume/weight in mice with mouse 4T1 breast cancer cells [63] and the human MCF7 breast cancer LA subtype [64]. The dark-phase fasting was found to delay HFD-induced mammary tumor onset and reduce HFD-induced tumor growth/weight in MMTV-PyMT mice [65], which are representative of the human LB subtype [66]. Interestingly, the FMD was not effective in reducing tumor progression in nude mice lacking T-lymphocytes [63], indicating the important role of the immune system in the beneficial effects of the fasting-mimicking condition in breast cancer. Our results using immunodeficient mice also exhibited less effectiveness of IF in the normal condition to decrease tumor volume and no significant change in the final tumor weight (Figure 5B,C). As IF improves immune function [14], the B6.129S7-Rag1tm1Mom/J strain used in this study may not have had enough immune activity for IF to damage the cancer cells because of its B and T cell deficiency compared to mice with a whole immune system.

Our in vitro and in vivo results showed that fasting conditions disrupted cell cycles and migration through cell cycle- and EMT-related proteins, affecting cell viability (Figure 2, Figure 3, Figure 4, Figure 7 and Figure 9). However, not all cancer cell types are susceptible to the fasting-mimicking condition. HCC827 (EGFR mutated non-small-cell lung cancer: NSCLC) and H3122 (ALK+ NSCLC) cells did not respond to the FM, whereas SKBR3 (HER2+ breast cancer), BT474 (HER2+ breast cancer), and HCT116 (colorectal cancer) cells were responsive to it, showing reduced cell viability [29]. Identifying fasting-sensitive and -resistant cancer cell types can help give adjuvant options for IF to treat cancer. Although H3122 (NSCLC) cells were not sensitive to the FM, fasting decreased tumor volume and weight in the H3122 cell line xenografts [29]. These findings indicate that IF systemically and locally affects cancer progression, emphasizing the important roles of the tumor microenvironment beyond direct effects on cancer cells.

Adipocyte CM decreased G1 and increased S and G2 phases in MCF7 cells [67]. The FM showed increased G1, decreased S, and G2/M phases in H3122 cells [29] and the human hepatic stellate LX-2 cell line [68]. These results suggest that the fasting-mimicking condition can directly target obesity-induced cell cycles in cancer progression. On the other hand, FM showed increased G1 and reduced the S phase by inhibiting cyclin D1 levels in human HepG2 cells despite having no effects on cell proliferation and motility [69], indicating an unknown gap in the fasting-mimicking condition between cell proliferation and cell cycles in a cell-type-specific manner. The FM-induced reduction in the S phase was observed in FM-treated TNBC MB231 and MB468 cells, but no significant change in the G1 phase (Figure 3C). In particular, an FM-induced increase in the G2 phase was detected in MB231 cells but not in MB468 cells, indicating the differential consequences of FM on cell cycles between mesenchymal-like and basal-like TNBC cells. Both MB231 and MB468 cells showed FM-induced downregulation of cyclin B1 (Figure 3C), which was supported by the in vivo results (Figure 9A). CCNB1 (cyclin B1 gene) was found to be overexpressed in TNBC and associated with TNBC progression, acting as a promising prognostic biomarker and therapeutic target for TNBC [70,71]. In addition, Aurora-A as a mitotic regulator was associated with recurrence and poor OS in obese patients with early breast cancer [72]. As Aurora-A interacts with cyclin B1, inhibiting cyclin B1 degradation [73], targeting the Aurora-A–cyclin B1 axis suggests promising new approaches for obesity-induced breast cancer prevention and treatment resistance [72]. Therefore, IF appears to be a promising strategy to target cyclin B1 in TNBC progression. FM-activated decreased cyclin E1 mRNA levels in MCF7 cells but had no effects on T47D and ZR-75-1 cells [64]. Although the FM-induced reduction in cyclin E1 and A2 proteins was detected in MB231 cells (Figure 3C), tumoral cyclin E1 and A2 levels were similar between HFD and HFD-IF (Figure 9A). These results indicate that complex mechanisms of cell cycles interact with other cell types in the IF-induced tumor microenvironment compared to FM-induced cell culture models.

The FM attenuated Erk activation in SKBR3, BT474, and HCT116 cells but had no effects on HCC827 and H3122 cells [29]. However, fasting conditions increased Erk activation in H3122 tumors despite decreased tumor volume and weight [29]. A study showed that the fasting condition decreased cell proliferation without altering Erk activation in human HepG2 cells [68]. The FM activated Akt in MCF7 and T47D cells but showed no effects in ZR-75-1 cells [64]. The FMD alone did not induce Akt activation in MCF7 cell tumors but decreased hormone-induced Akt activation [64]. Our results showed that the effectiveness of the FM was minimal on survival and proliferative signaling pathways in TNBC cells (Figure 4A). These differential effects of fasting conditions on survival- and proliferation-related proteins such as Akt and Erk reflect cell-type and fasting-type specific manners. Autophagy, which ensures cellular homeostasis through the recycling of unnecessary components, can suppress or promote tumors in the developmental stage in a tumor-type-dependent manner [15]. Although fasting is known to improve autophagy-associated proteins [74], the FM’s effect was minimal on autophagy-related proteins in TNBC cells (Figure 4B).

Adipocyte CM enhanced the wound closure assay and cell invasion by increasing EMT-related protein levels, such as vimentin, snail, and twist [67], indicating an obesity-induced EMT change. The FM targeted EMT-related proteins vimentin and β-catenin in TNBC MB231 and MB468 cells, respectively (Figure 4C,D). Wnt/β-catenin signaling was particularly activated in TNBC, serving as a novel therapeutic target for the treatment of TNBC [75]. A Wnt-driven lung metastasis model of TNBC metM-Wntlung cells showed enhanced metastasis in obese mice compared to non-obese mice [76], implying obesity-induced EMT. Elevated glucose levels increased β-catenin expression in endometrial cancer cells [77], supporting the downregulation of the FM with low glucose and β-catenin levels in MB468 cells (Figure 4C,D). Furthermore, an FM-induced reduction in vimentin in mesenchymal-like TNBC MB231 cells was confirmed in mouse mesenchymal-like TNBC PY8119 cells (Figure 4C) and MB231 cell-derived tumors (Figure 7E). LNCaP, PacMetUT1, and DU145 prostate cancer cells exposed to obese sera resulted in increased invasion and migration, showing increased vimentin [78]. An adipocyte CM-induced invasion of B16BL6 melanoma cells was associated with an increased expression of EMT genes such as snail, twist, and vimentin mRNA levels [79]. The reverse-phase protein microarray revealed that vimentin expression was significantly increased in mammary epithelial cells of obese women compared to those of non-obese women [80], appearing as an obesity-sensitive EMT protein. Accordingly, silencing vimentin expression decreased pulmonary metastases in a pre-diabetic mouse model of mammary tumor progression [81].

IF reduced HFD-induced blood glucose and total cholesterol but had no effects on triglyceride levels in obese rats [62], being consistent with our results (Figure 6). FMD decreased blood glucose levels in patients with BC in the NCT03595540 and NCT03340935 clinical trials [64]. A systematic review showed that the fasting condition was associated with a significant decline in BMI and glucose levels [82]. Cancer cells show enhanced glucose uptake and the conversion of a significant amount of glucose into lactic acid, even in the presence of oxygen, the so-called Warburg effect [83]. An IF-induced reduction in plasma glucose levels might be more critical to the survival of cancer cells rather than normal cells. Although high blood cholesterol is a common comorbidity in obesity [84], cholesterol as a risk factor in breast cancer is still controversial, and whether total, LDL, or HDL cholesterol contributes to breast cancer is unclear [85]. Further study is required to establish the consequences of IF-induced cholesterol reduction in TNBC progression.

Our previous studies showed that Mφ was highly accumulated in ovarian omental tumors and ascites from obese mice compared to those from lean mice [32,86]. Obesity induces mammary white adipose tissue expansion and is coupled with an accumulation of immune cells, specifically Mφ, followed by a worse prognosis for breast cancer [87]. IF used against obesity could contribute to decreased Mφ accumulation in the obesity-associated tumor microenvironment (Figure 8C), probably decreasing chronic inflammation followed by the potential suppression of TNBC progression. The HFD increased immune suppressive MDSC in the blood, limiting the activation of tumor-reactive CD8+ T cells, and the HFD-induced tumor microenvironment showed elevated levels of IL-1β and IFNγ mRNA levels in 4T1 cell-bearing mice [88]. HFD-induced obesity enhanced the accumulation of MDSCs in the tumor microenvironment of pancreatic tumor-bearing mice, reducing the CD8 T cell:MDSC ratio [89]. Although IF showed no effects on the accumulation of MDSCs in the tumor microenvironment (Figure 8D), it reduced proinflammatory factors C5, IL-1β, CCL3, and CXCR2 in the HFD-induced tumor microenvironment (Figure 9B). HFD-induced obesity promoted the accumulation of PD-1+ CD8+ exhausted T cells in tumors of MMTV-PyMT mice [90], probably decreasing anti-tumor cytotoxic activity. The FMD showed no change in tumor-infiltrating CD4 and CD8 cells in 4T1 cancer cell-bearing mice [63]. Because B and T cell-deficient mice were used in this study, further study is required to clarify if IF affects tumoral CD4 and CD8 infiltration in TNBC using intact mice. The FMD significantly inhibited chemotherapy-induced DNA damage in T-lymphocytes [91], reducing hematological toxicity. Therefore, IF emerges as a promising strategy to enhance the efficacy and tolerability of chemotherapy [92]. However, severe fasting or calorie restriction causes a drastic reduction in the number of monocytes and lymphocytes in the blood and in peripheral organs [93]. Thus, an adequate balance between feeding and fasting is needed to maximize the anti-tumor immune response. As obesity shapes metabolism in the tumor microenvironment to suppress anti-tumor immunity [94], a correct fasting strategy may reprogram the obesity-induced metabolism in the tumor microenvironment to improve anti-tumor immunity and enhance current therapeutics. The local and systemic inflammation of white adipose tissue in the breast has been associated with an increased risk of breast cancer [95] and poor outcomes for women with breast cancer [96]. Accordingly, an IF-induced reduction in proinflammatory factors in the obesity-derived tumor microenvironment may help attenuate obesity-induced TNBC progression.

Although the HFD-induced obesity model in mice cannot entirely reflect the obese human population under various factors, it may provide a routine and efficient alternative for the study of the obese human population, enabling the exploration of molecular mechanisms in obesity-related cancers. Because IF is more effective in obese mice rather than non-obese mice (Figure 5A), it may play a critical role in the obese population with obesity-related cancers [3]. In addition, since many patients with cancer lose weight at some point in cancer progression, further studies require developing a clinical application to improve the benefits of IF for treating cancer. Also, metabolic aspects of IF may be valuable to target obesity-induced TNBC progression as a future perspective.

In summary, HFD-induced obesity boosts body weight and blood glucose/cholesterol to create an obesity-associated microenvironment for TNBC, leading to an increased tumoral cell cycle, EMT, and Mφ accumulation, followed by accelerated TNBC progression and mortality (Figure 9C, red arrows). On the other hand, IF targets the obesity-induced parameters, lowering body weight and blood glucose/cholesterol to destroy the obesity-associated microenvironment of TNBC with downregulated cyclin B1/vimentin and Mφ accumulation followed by decelerated TNBC progression and mortality (Figure 9C, green arrows).

## 5. Conclusions

IF can attenuate obesity-induced TNBC progression through reduced obesity and tumor burdens, disrupting cell cycles, EMT, and tumor immune contexture. Lifestyle intervention like IF may provide optimistic prospects to improve prognosis and survival in obesity-associated TNBC, reducing obesity and potentially, the mortality burden of TNBC. Additional evidence is needed to validate these potential benefits in patients with TNBC, including through human clinical trials.

## Data Availability

The data used to support the findings of this study are available upon reasonable request from the corresponding author.
